# Coagulation Factor Xa Promotes Solid Tumor Growth, Experimental Metastasis and Endothelial Cell Activation

**DOI:** 10.3390/cancers11081103

**Published:** 2019-08-02

**Authors:** Maximiliano Arce, Mauricio P. Pinto, Macarena Galleguillos, Catalina Muñoz, Soledad Lange, Carolina Ramirez, Rafaela Erices, Pamela Gonzalez, Ethel Velasquez, Fabián Tempio, Mercedes N. Lopez, Flavio Salazar-Onfray, Kelly Cautivo, Alexis M. Kalergis, Sebastián Cruz, Álvaro Lladser, Lorena Lobos-González, Guillermo Valenzuela, Nixa Olivares, Claudia Sáez, Tania Koning, Fabiola A. Sánchez, Patricia Fuenzalida, Alejandro Godoy, Pamela Contreras Orellana, Lisette Leyton, Roberta Lugano, Anna Dimberg, Andrew F.G. Quest, Gareth I. Owen

**Affiliations:** 1 Faculty of Biological Sciences, Pontificia Universidad Católica de Chile, Santiago 8331150, Chile; 2 Advanced Center for Chronic Diseases (ACCDiS), Santiago, Chile; 3 Faculty of Medicine, Pontificia Universidad Católica de Chile, Santiago 8331150, Chile; 4 Vicerrectoría de Investigación, Universidad Mayor, Santiago 7510041, Chile; 5 Comisión Chilena de Energía Nuclear (CCHEN), Santiago, Chile; 6 Institute of Biomedical Sciences, Faculty of Medicine, University de Chile, Santiago 8380453, Chile; 7 Millennium Institute on Immunology and Immunotherapy, Santiago 8331150, Chile; 8 Biomedical Research Consortium of Chile, Santiago 8331010, Chile; 9 Laboratory of Immunoncology, Fundación Ciencia & Vida, Santiago, Chile; 10 Regenerative Medicine Center, Faculty of Medicine, Clinica Alemana-Universidad Del Desarrollo, Santiago 7650568, Chile; 11 Immunology Institute, Faculty of Medicine, Universidad Austral de Chile, Valdivia 5110566, Chile; 12 Department of Urology, Roswell Park Comprehensive Cancer Center, Buffalo, NY 14203, USA; 13 Laboratory of Cellular Communication, ICBM, Faculty of Medicine, Universidad de Chile, Santiago 8380453, Chile; 14 Department of Immunology, Genetics and Pathology, Rudbeck Laboratory, Uppsala University, 751 85 Uppsala, Sweden

**Keywords:** cancer, metastasis, melanoma, blood coagulation, vascular endothelium, inflammation

## Abstract

Hypercoagulable state is linked to cancer progression; however, the precise role of the coagulation cascade is poorly described. Herein, we examined the contribution of a hypercoagulative state through the administration of intravenous Coagulation Factor Xa (FXa), on the growth of solid human tumors and the experimental metastasis of the B16F10 melanoma in mouse models. FXa increased solid tumor volume and lung, liver, kidney and lymph node metastasis of tail-vein injected B16F10 cells. Concentrating on the metastasis model, upon coadministration of the anticoagulant Dalteparin, lung metastasis was significantly reduced, and no metastasis was observed in other organs. FXa did not directly alter proliferation, migration or invasion of cancer cells in vitro. Alternatively, FXa upon endothelial cells promoted cytoskeleton contraction, disrupted membrane VE-Cadherin pattern, heightened endothelial-hyperpermeability, increased inflammatory adhesion molecules and enhanced B16F10 adhesion under flow conditions. Microarray analysis of endothelial cells treated with FXa demonstrated elevated expression of inflammatory transcripts. Accordingly, FXa treatment increased immune cell infiltration in mouse lungs, an effect reduced by dalteparin. Taken together, our results suggest that FXa increases B16F10 metastasis via endothelial cell activation and enhanced cancer cell-endothelium adhesion advocating that the coagulation system is not merely a bystander in the process of cancer metastasis.

## 1. Introduction

The first observation linking exacerbated thrombus generation in cancer patients came from Armand Trousseau in 1865. Cancer presence has been related to coagulation cascade promotion and platelet activation, which can impinge tumor biology and generates a chronic hypercoagulable state in patients [1]. As a consequence, venous thrombosis (VT) is the second leading cause of death among cancer patients worldwide [2,3]. Moreover, there is an association of high D-dimer levels (final product of the coagulation cascade activation) with advance cancer stage and poor prognosis in melanoma patients [4]. Furthermore, a high prevalence of venous thromboembolism in stage IV melanoma patients was found in a retrospective study [5], showing similar percentage of affected patients than in lung and gastrointestinal cancer (both with high prevalence of VT). Tissue factor (TF) is a membrane protein overexpressed in several tumor types, when in contact with blood initiates the extrinsic pathway of the coagulation cascade by binding to Coagulation Factor VIIa (FVIIa) [6,7]. The complex TF-FVIIa promotes the formation of Coagulation Factor Xa (FXa) via proteolytic cleavage and the sub consequently initiation of the common pathway of the coagulation cascade [8]. Interestingly, chemotherapeutic drugs can stimulate the activation of the coagulation cascade by increasing TF levels or by decreasing Tissue Factor Protein Inhibitor-1 (TFPI-1), a protein that specifically binds TF acting as an inhibitor of the proteolytic activity of the TF-FVIIa complex [9]. Additionally, other mechanisms have been proposed associated to chemotherapy-procoagulant effect, including cellular stress and apoptosis of endothelial cells [10]. Isolation of platelet and endothelial-derived microvesicles from melanoma patients showed a procoagulant potential compared to healthy subjects, suggesting an association of cancer presence with thrombogenesis [11].

Coagulation factor X (FX) plays a central role in the coagulation cascade. Synthesized in the liver, FX and can be proteolytically activated to form FXa. Activation may occur via the extrinsic or the intrinsic coagulation pathways. Once activated FXa unleashes a proteolytic cascade that converts prothrombin into thrombin, leading to the formation of fibrin networks, platelet activation eventually generating a blood clot. Besides their role in the coagulation cascade, both FXa and Thrombin can also act as activators of Protease Activated Receptors (PARs) triggering different responses in several cell types such as: fibroblasts, platelets, endothelial and cancer cells [12,13]. Accordingly, FXa via PAR-1 activation can inhibit breast, colon and lung cancer cell line migration [14]. In endothelial cells, FXa and thrombin increase the expression of pro-inflammatory molecules such as interleukin 6 and 8 (IL-6 and IL-8) [15]. The relation between the coagulation pathway and inflammation have been addressed extensively, in which thrombin (independent of FXa) appears to be the key molecule in this association [16].

Hematogenous metastasis is a highly ordered, stepwise process that starts when cancer cells acquire an invasive phenotype, detach from the primary tumor and enter the bloodstream in a process termed hematogenous intravasation [17]. The cancer cells subsequently can migrate to distant organs and establish metastatic foci after extravasation from the bloodstream. Extravasation first requires the attachment and firm adhesion of the cancer cell to the endothelium. This process is mediated principally by integrins and selectins expressed both in the cancer and on the endothelial cell [18,19,20]. Endothelial selectins, such as: P-selectin, E-selectin, and the intercellular adhesion molecules: Intercellular Adhesion Molecule 1 (ICAM-1), Vascular cell adhesion molecule 1 (VCAM-1) and their ligands, have been related to immune cell and cancer cell adhesion to the vasculature [21]. After firm adhesion, cancer cells must transverse the highly selective endothelial barrier. The permeability of the endothelial barrier is principally maintained by adherence junctions and occludines, of which the most studied proteins is vascular endothelial cadherin (VE-cadherin) [22]. Under inflammatory conditions, VE-cadherin complex disassembles [23], promoting vascular hyperpermeability and allowing the extravasation of immune cells into the target tissue. Cancer cells can exploit this physiological process to facilitate metastasis [24]. Interestingly, in vitro observations showed that thrombin promotes endothelial hyperpermeability through the activation of the small GTPase RhoA (Ras homolog gene family member A) [25].

Previous reports suggest that direct oral anticoagulants (DOACs, specific protease inhibitors of the coagulation cascade) increase survival rates among cancer patients [26]. In contrast, the *FRAGMATIC* trial found that adjuvant dalteparin (a low molecular weight heparin anticoagulant) had no effect upon survival rates in advance lung cancer patients [27]. Despite the negative results with dalteparin, a similar trial found that its use in combination with chemotherapy increased survival rates among lung small-cell carcinoma patients (compared to patients that only received chemotherapy) [28]. Significantly, a twelve-month treatment with dalteparin in a multiple-cancer clinical trial showed an increase in the overall survival in a subgroup of patients with better prognosis, suggesting a potential role of this drug over metastasis and tumor biology [29]. 

The use of anticoagulants in cancer patients is still controversial and their use as an adjuvant treatment is under debate, in part due to the potential side effects such as thrombocytopenia and spontaneous hemorrhage. The current information suggests that the coagulation system may not be merely a bystander in the process of cancer metastasis. The hypercoagulable state observed in many cancer patients, not only increases the risk of thrombosis, but may alter endothelial barrier properties and thus promote cancer cell metastasis.

Taken together, clinical and in vitro studies suggest a contribution of the coagulation system to inflammation and metastasis, however the mechanisms are still elusive. Several in vivo models have been stablished to study importance of the coagulation cascade and the relation to other diseases [30]. Furthermore, the anti-inflammatory effect of human-FVIIa has been demonstrated through intravenous administration in mice [31], suggesting an independent role of coagulation factors over inflammatory response. Herein we demonstrate that increasing FXa in circulation promotes cancer growth and suggest that enhanced metastasis occurs through endothelial activation.

## 2. Materials and Methods

### 2.1. Reagents and Cell Culture

FX, FXa and Factor Xa EGRck active site blocked (HCXA-EGR) were purchased from Haematologic Technologies (Essex, VT, USA). Antibodies: VCAM-1 and ICAM-1 (Santa Cruz, CA, USA), pFAK (Abcam, San Francisco, CA, USA), VE-cadherin (Cell Signaling, Danvers, MA, USA). Matrigel Growth Factor Reduced (354230, Corning, Painted Post, NY, USA) and phalloidin-rhodamine (ThermoScientific, Carlsbad, CA, USA) were purchased from local distributors and dalteparin 2500UI (Pfizer, New York, NY, USA) was purchased directly from a local pharmacy. The endothelial cell line EAhy926 (CRL-2922) and the melanoma cell line B16F10 (CRL-6475) cells were purchased from the ATCC (Manassas, VA, USA). The human melanoma cell line Mel-1 was kindly donated by Dr Lopez and Dr. Salazar (University of Chile), Ishikawa cells by Dr. John White (Hammersmith Hospital, London, UK) and HPMEC-1 by Dr. Claudia Sáez (Catholic University of Chile). Primary cultures of Human Umbilical Vein Endothelial Cells (HUVECs) were isolated from fresh umbilical cords and obtained as previously described [32]. All procedures were approved by the Ethics Committee of Pontifical Catholic University of Chile. All cells were cultured at 37 °C, 5% CO_2_ in Dulbecco's Modified Eagle Medium (DMEM)-F12, Iscove's modified Dulbecco's medium (IMDM), growth medium MV supplemented with endothelial growth factors or serum-free media (SFM) supplemented with endothelial cell growth factors and 10% Fetal bovine serum (FBS) (S1810, Biowest, Nuaillé, France). 

### 2.2. Flow Cytometry

Cell cycle and chemokine receptor analysis was performed as previous described [33]. Briefly, cells were treated with FXa or vehicle (water) for 24 h, then fixed with cold 70% ethanol and incubated with propidium iodide (0.5 mg/mL). Flow cytometry was performed using Fluorescence Activated Cell Sorter (FACScan system) and the Cell-Quest program (Beckton-Dickinson, Covington, GA, USA). For the analysis of chemokines receptor expression in B16F10, cells were treated with FXa for 24 h and harvested in trypsin solution. Then cells were washed with PBS/FBS 2% stained with antibodies for flow cytometry for 30 min: L-selectin (αCD62L), Chemokine receptor CXCR3 (αCXCR3), Chemokine (C-C motif) receptor 8 (αCCR8), C-C chemokine receptor type 7 (αCCR7) and Zombie-aqua for cell viability). Cells were washed twice before analysis. 

### 2.3. Regression Assay

EA.hy926 and HUVECs (30,000 cells) were seeded onto 48 well plates pre-coated with non-diluted Matrigel and tubular-like structure formation was promoted by Vascular Endothelial Growth Factor (VEGF, Fisher Scientific, Madrid, Spain) 10 ng/mL (in serum free media) for eight hours. FX(a) was added and images were taken at 0, 2 and 4 h. An angiogenesis score was calculated using the analyzer plugin from ImageJ (NIH, Bethesda, MD, USA). Master segments were quantified and expressed as the percentage of linked networks, assuming 0 h as a 100%. FX, FXa and HCXA-EGR were added at 130nM. PAR-1 TFLLRN (H-Thr-Phe-Leu-Leu-Arg-NH2) and PAR-2 SLIGKV (H-Ser-Leu-Ile-Gly-Lys-Val-NH2) agonists were obtained from Peptides International (Louisville, KY, USA). Agonists were applied at a concentration of 10 micromolar in aqueous solution.

### 2.4. In Vitro/In Vivo Permeability Assay

EA.hy926 cells were grown to 100% confluent monolayer onto 3 µm pore Transwell inserts (PITP01250, Merck-Millipore, Watford, UK) pre-coated with 1% gelatin and permeability analyzed by Evans Blue [34]. Animal protocols were approved by the Institutional Bioethics and Biosecurity Committee at the Universidad Austral de Chile and conducted according to NIH Guideline. Microvascular permeability to macromolecules was measured by intravital microscopy in the mouse cremaster muscle according to published methods [35,36]. Briefly, C57BL/6 male mice (30–50 g) were anesthetized with ketamine (80 mg/kg) and xilazine, (8 mg/kg) intraperitoneal (i.p); and the left cremaster muscle was prepared for intravital microscopy, by exposing the tissue and placing it in an ad hoc observation chamber, under constant perfusion with bicarbonate buffer as described. Fluorescein isothiocyanate (FITC)-Dextran 70 KDa (100 mg/kg) was injected intravenously (i.v) as a fluorescent macromolecular tracer. Plasma to tissue transport was evaluated in situ by integrated optical intensity (IOI). FXa was added topically in the cremaster for 15 min. At the end of the experiment, the animal was sacrificed by an anesthetic overdose (~300 mg/Kg i.v.), followed by pneumothorax.

### 2.5. Immunofluorescence

HUVECs cells were grown to high/low confluence state on glass coverslips. Then treated with FXa (20 and 130 nM) in serum free media and fixed with 4% paraformaldehyde (PFA) at room temperature. Cells were then washed with 1× PBS and incubated with PBS-Triton 0.1% for 5 min and blocked with 2% Bovine Serum Albumin and PBS (BSA-PBS) for 30 min at room temperature. Primary antibody was incubated overnight in humid chamber at 4 °C and the secondary antibodies (Anti-rabbit Alexa Fluor 488 and 555) and phalloidin-rhodamine were incubated for one hour at room temperature. Cell nuclei were stained by 4′,6-diamidino-2-phenylindole (DAPI) and coverslips were mounted using Fluoromount (1798425, Fisher Scientific, Madrid, Spain). Images were obtained from independent fields for each coverslip in a C2s1 microscope (Nikon, Melville, NY, USA) and processed/analyzed using ImageJ software (NIH, Bethesda, MD, USA). To evaluate the localization and recruitment of the active form of RhoA in response to FXa, HUVECs were treated in serum free media for 5 min, fixed with Trevors fixation buffer and maintained in universal buffer until beginning of the immunofluorescence [37]. Cells were permeabilized with Triton 0.1% and blocked using BSA 2% for 30 min. Cells were incubated with anti RhoA-GST cassette (dilution 1:25) for 2 h at room temperature in humid chamber. Anti-GST (1:100), anti-mouse Alexa Fluor 488 (Thermo Fisher Scientific, Carlsbad, CA, USA) (1:300) and phalloidin-rhodamine (1:500) were incubated for 1 h at room temperature in humid chamber. Cells were mounted with Mowiol embedding medium and visualized using epifluorescence microscopy (Nikon C2s1, Melville, NY, USA).

### 2.6. Underflow and Static Adhesion Assay

HUVECs were seeded in a gelatin-coated microchannel slide (ibiTreat µSlide Luer I^0.4^; Ref. 80176, Ibidi, Gräfelfing, Germany) and cultured until confluence in Endothelial Cell Basal Medium with full supplements (EBM-MV2, PromoCell, Heildeberg, Germany) at 37 °C and 5% CO_2_/95% air in a humidified chamber. B16F10 cells (1.7 × 10^6^ cells/mL) were pumped into microchannel slide under 50 uL/min flow speed using a syringe pump (NE-1200X; New Era Pump System Farmingdale, NY, USA) enabling B16F10 cells to interact with the monolayer of control or FXa pre-treated HUVEC. Adhesion of B16F10 cells to the endothelial monolayer was monitored using a SP8 microscope (Leica, Allendale, NJ, USA) equipped with a motorized xyz stage and with a temperature-controlled CO_2_-incubation system for live cell time-lapse imaging. Differential interference contrast (DIC) images of three distinct areas along the microchannel were taken using 10× objective every 10 s during a total of 25 min. The number of B16F10 cells adhering to the HUVEC monolayer was quantified by ImageJ software.

For the static adhesion assay, HUVEC were plated and grown to 100% confluence on a 48-well plate. Cells were treated with FXa for 4 h. B16F10-eGFP cells (10,000) were seeded over an endothelial monolayer for 30 min. The well was washed with warm 1x PBS to remove unattached B16F10-eGFP cells, before fixing for 20 min with PFA 4%. Visualization was by epifluorescence microscopy Nikon C2s1 (Melville) and quantification by ImageJ software (NIH). 

### 2.7. Cell Migration and Invasion

For the scratch assay (wound healing assay), B16F10 confluent 6-well plates were scratched with a yellow pipette tip, before washing and treatment with FXa or vehicle in serum free medium. The plate was photographed at varying time points and the area of wound closure was quantified with ImageJ. Cell invasion was analyzed by Transwell inserts (8 μm pore size, Merck-Millipore, Watford, UK) as described previously [38]. Briefly, B16F10 cells (50,000) were seeded over the Matrigel (1:5 dilution in serum free media) and 800 μL of 10% of serum medium was added to the lower chamber as a chemoattractant. FXa (130 nM) was added in the upper chamber together with cells for 8 h. Transwells were fixed with warm 4% PFA for 20 min at room temperature and stained with crystal violet for 10 min. The membranes were removed and mounted on cover slides with warm 10% for counting. 

### 2.8. Western Blot

Proteins were quantified by the Bradford method. After gel electrophoresis and transfer, the membranes were blocked with TBS-Tween 5% Milk and primary antibodies (1:500 dilution, overnight at 4 °C). The secondary antibody (anti rabbit/mouse-horseradish peroxidase 1:3000 dilution) was incubated for 1 h at room temperature before visualization using the myECL system (ThermoFisher, Carlsbad, CA, USA).

### 2.9. Metastasis and Tumor Growth In Vivo Experiments

All protocols and animal management were performed in accordance and approval with the ethics and bioethics committees of the respective institutions. All subjects gave their informed consent for inclusion before they participated in the study. The study was conducted in accordance with the Declaration of Helsinki and international animal guidelines with approval from the ethical committee of the Ministry of Health Central Metropolitan Region No. ESR/GGN/JEC No. 579/14 (date 15th July 2014), the bioethics committee of University of Chile No. CBA0642FMUCH (date 14th October 2013), the bioethics committee of Fundación Ciencia & Vida S/N (date 11th April 2017) and the ethics committee No. 13-226 CBB-186 (dates 8th January 2013 and 3rd September 2013) and bioethics committee No. CBB-186/2013 (date 23rd September 2013) of the Pontifical Catholic University of Chile. Analysis of hypercoagulative state through the administration of intravenous coagulation factors has been reported previously [31]. Animal studies using Ishikawa endometrial cells in NIH nu/nu mice (purchased from the Universidad Nacional de La Plata, Buenos Aires, Argentina) were performed at the animal facility of the Pontifical Catholic University of Chile. Mel-1 melanoma studies were performed in Non-obese diabetic/severe combined immunodeficiency (NOD/SCID) mice purchased from the animal facility at the University of Chile. For the analysis of solid tumors, Ishikawa cells (5 × 10^6^ cells) or Mel-1 cells (2 × 10^6^ cells) were injected subcutaneously into the flank of the immunocompromised female NIH nu/nu mice and NOD/SCID mice respectively. Before initiating the experimental protocol, we tested whether intravenous administration of coagulation factors presented detrimental effects. No fatalities, notable thrombus formation or vein occlusion were observed upon coagulation factor injection. FXa, FX or vehicle (PBS) was added intravenously every 2 days for 21 days. B16F10 melanoma cells (200,000) were injected (in 400μL of sterile saline solution) into the tail vein of 7–9 week C56BL/6 mice at the animal facility of Andes Biotechnologies within the Science and Life Foundation, Santiago, Chile. FXa was added at a circulatory concentration of 130 nM into the tail vein twice a week for up to 21 days. Dalteparin at a final concentration of 200 UI/kg (diluted in 150 µL of saline solution) was injected subcutaneously 30 min before FXa administration. Animals were sacrificed and autopsied before maximum permitted tumor size was reached.

#### 2.9.1. Microarray Analysis

Endothelial cells (EA.hy926) were treated with FXa 130 nM by 4 h in serum free media. The RNA extraction was performed using an RNA isolation kit. A total of 6 samples were assayed on Affymetrix whole transcriptome expression (HuGene-2_0-st-v1, Affymetrix NIH, ThermoFisher, Carlsbad, CA, USA) in which 53.617 transcripts were analyzed. The data was processed at the Microarray Facility at The Centre for Applied Genomics (Sick Kids Hospital, Toronto, ON, Canada). All steps were performed with the statistical analysis program R version 3.2.0 (https://cran.rproject.org/), using the Bioconductor package oligo version 1.32.0. Once the samples were filtered, the Differential Expression Analysis was carried out, contrasting the two study groups, using Linear Models for Microarray Analysis, Limma (Bioinformatics Division, The Walter and Eliza Hall Institute of Medical Research, Melbourne, Australia). A Fold Change (FC) = 1 and a *p* value < 0.05 were established, thus obtaining 127 overexpressed genes and 37 genes were down-regulated in the cells. The algorithm of Benjamini and Hochberg was used to adjust the *p*-value and eliminate the false positives from the analysis. Data analysis was performed in the CORE biodata facility of the Advance Center for Chronic Diseases (ACCDiS, Santiago, Chile).

#### 2.9.2. Statistical Analysis

For the comparison of the data obtained between treatments, an analysis of variance (ANOVA) or *t*-test statistical analysis was performed followed by a Bonferroni post-test, considering a value of *p* < 0.05 as statistically significant. For the in vivo experiments a minimum of 3 animals were used per group.

## 3. Results

Given the clinically-suggested association between a hypercoagulable state and cancer progression, we wished to determine if alterations in the coagulation system changed tumor growth and metastasis. Models to study importance of the coagulation and disease progression often use vein occlusion as the promoter of the coagulation cascade and thrombus formation [30]. While several models of hypercoagulable state exist, the administration of intravenous coagulation factors in mouse models has been reported previously to study inflammation and thus we extrapolated this model to the setting of cancer progression [31]. The physiological concentration of zymogen FX is reported to be in the range of 130 nM [39]. Experimentation in vitro has suggested that the concentration of FXa generated from tissue factor (TF) microvesicles and acting upon a substrate is in the range of 10–50 nM. While the zymogen has a half-life of 40 h, FXa half-life is only believed to be minutes due to inactivation by circulating serine protease inhibitors, such as antithrombin [40]. Thus, in our animal models we added a higher concentration (130 nM) of FXa to have a prolonged enzymatic effect and sites distance to the tail vein injection (the animal flank for the solid tumors and upon the lungs as B16F10 produces primarily pulmonary metastasis). The concept of FXa intravenous introduction, applied in the study presented herein, was to create a hypercoagulable environment in a healthy animal and preliminary experiments with this concentration of FXa demonstrated that this dose was well tolerated, with no adverse side effects or animal deaths being observed.

### 3.1. FXa Increased Metastasis In Vivo, An Effect Inhibited by Dalteparin Treatment

**Figure 1:**
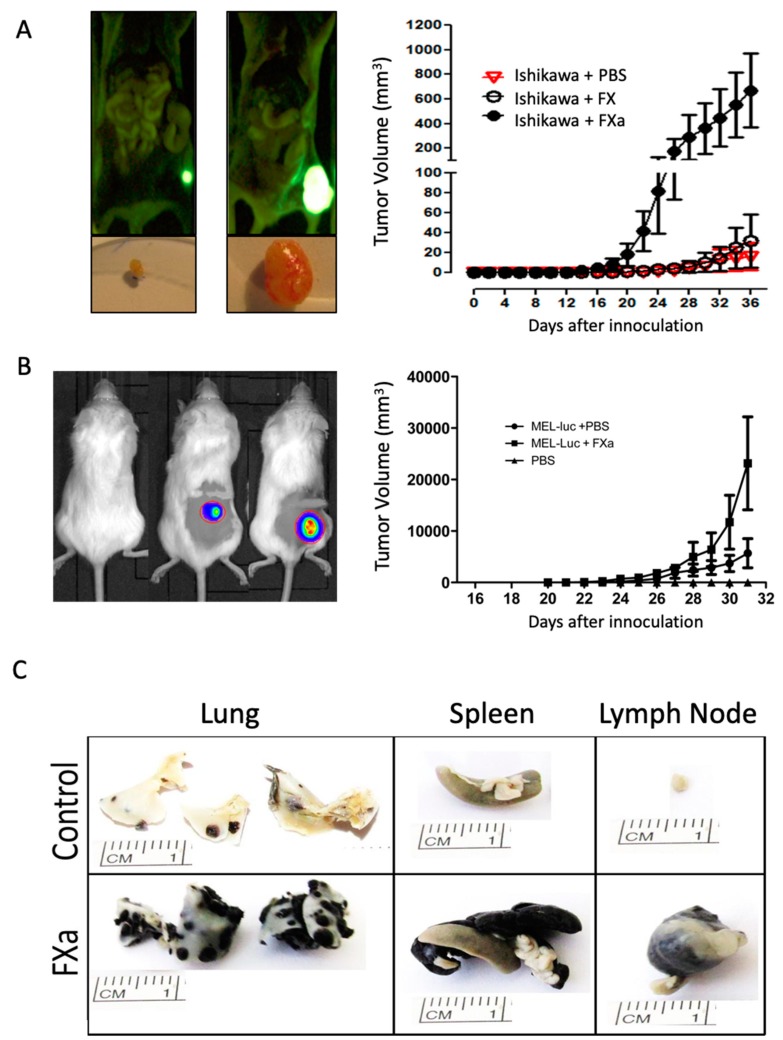
Coagulation Factor Xa increases solid tumor volume and experimental metastasis. (**A**) Human endometrial cancer (Ishikawa-ZsGreen) and (**B**) human melanoma cells (Mel-1-Luciferase) were injected subcutaneously in the flank of National Institute of Heath nude (NIHnu/nu) and Non-obese diabetic/severe combined immunodeficiency (NOD/SCID) mice respectively. FXa (at 130 nM as a final concentration) was administrated intravenously every two days throughout the experiment. Tumor volume was determine using calipers in the endometrial cancer model and IVIS chemiluminescence and calipers in the melanoma model. The equation Tumor Volume (mm^3^) = (long × width^2^)/2 was used and shown in both models. *n* = 4, *p* < 0.05 analysis of variance (ANOVA). (**C**) B16F10 cells (200,000) were injected into the tail vein of C57BL/6 mice and FXa (or vehicle, control) was administrated intravenously twice a week until euthanasia and autopsy at 21 days.

To evaluate the effect of increased circulating FXa levels, Ishikawa human endometrial cancer cells were subcutaneously introduced into the flank of immunosuppressed NIH nu/nu mice and the human melanoma cell line Mel-1 into the flank of NOD/SCID mice. Mice were treated with FXa or vehicle intravenously three times a week until the end of experimentation. As shown Figure 1, intravenous FXa increased the solid tumor mass of both the endometrial cancer model and the human melanoma cell tumor (Figure 1A,B). No metastases were observed upon autopsy in either model. Recombinant zymogen FX did not influence tumor growth. To determine if this effect of FXa was not restricted to solid tumors, we examined the treatment of this activated coagulation factor on the process of metastasis. B16F10 cells were injected into the tail vein of C57BL/6. Mice were treated with FXa or vehicle intravenously three times a week until they completed 21 days of experimentation. Representative images of the lung metastasis are shown (Figure 1C), showing an increased tumor burden in the FXa group. Notably, metastasis outside of the lungs were observed only in mice treated with FXa (Figure 1C). Zymogen Factor X did not increase solid tumor growth or metastasis (Figure 1). 

While three separate cancer cell models demonstrated either notably increased growth or metastasis, clearly stating a correlation with hypercoaguable state and cancer progression, we decided to concentrate on the association between metastasis and coagulation observed in our syngeneic model of B16F10 melanoma in C57BL/6 mice. 

**Figure 2:**
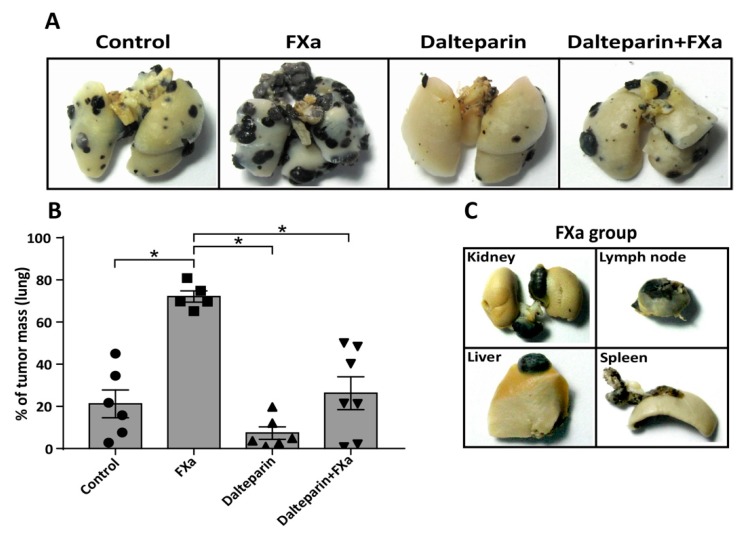
Coagulation Factor Xa promotes melanoma metastasis, an effect reduced by Dalteparin. (**A**) 200,000 B16F10 cells were injected into the tail vein of C57BL/6 mice and FXa (or vehicle, control) was administrated intravenously twice a week until euthanasia at 21 days. The anticoagulant dalteparin was injected sub-cutaneously 30 min before FXa administration. Representative images from metastasis-affected lungs are shown. (**B**) Quantification of lung tumor burden was assessed through the weighing of total lung mass and the dissected tumor mass. * Statistically significant difference *p* < 0.05 analysis of variance (ANOVA), post-test Bonferroni. (**C**) Representative images of organ metastasis found exclusively in mice from the FXa group.

Repeating the experiments from Figure 1C and adding an anticoagulant treatment (with the low molecular weight heparin dalteparin, 200 UI/kg, which preferentially inhibits FXa) in combination with FXa injections we observed that dalteparin inhibited the pro-metastatic effect of FXa in this mouse model (Figure 2A,B). The effect of the zymogen FX injection showed no statistical differences compared to the control group. Notably, liver, kidney, spleen and lymph nodes metastasis were once again observed only in mice treated with FXa (Figure 2C) and dalteparin treatment prevented the appearance of metastasis outside the lung. 

In search of a mechanism by which FXa increases metastasis, we first hypothesized that FXa has an effect upon cancer cells. As shown in Supplementary Figure S1, FXa addition (130 nM) did not alter B16F10 cell migration (Figure S1A,B). Moreover, FXa treatment did not alter cell death (appearance of a sub G1 peak by flow cytometry) or bring about significant changes in the cell cycle (Figure S1C). The lack of changes in proliferation was further confirmed using the cell proliferation reagent WST-1/viability assay (CELLPRO-RO, Merck, Watford, UK) (Figure S1D). Flow cytometry analysis confirmed that FXa did not change the levels of the cytokine receptors: CCR7, CCR8, CXCR3 and CD62L (Figure S1E) which may have been related to differential homing of the cancer cells observed in Figure 1C in the presence of FXa. Furthermore, invasion capabilities were not affected in response to FXa (Figure S1F). Additionally, we tested whether FXa could stimulate pro-angiogenic factors when in contact with cancer cells, however the conditioned medium from cancer cells treated with FXa did not alter capillary-like formation on the endothelial cells. To demonstrate that the lack of an FXa affect was not limited to B16F10 cells we repeated experiments in the human Ishikawa and Mel-1 cell lines. Once again, as observed in Supplementary Figure S1, no change in either cell cycle (panels G and H) or migration (panel I) was observed. 

### 3.2. FXa Alters Endothelial Cell Morphology and Contractibility

**Figure 3:**
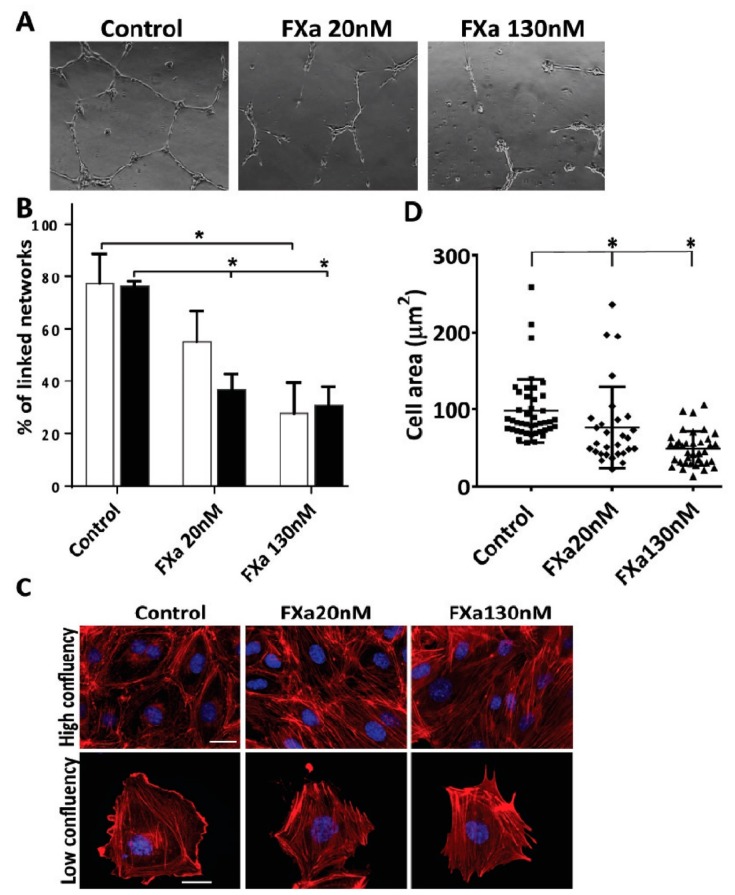
Coagulation Factor Xa disrupts tubular structures on Matrigel and promotes endothelial cell contraction. (**A**) Human Umbilical Vein Endothelial Cells (HUVECs) were seeded in Matrigel in the presence of Vascular Endothelial Growth Factor (VEGF) (10 ng/mL). 15 h later, and on the tubular structures already formed, FXa in two different concentrations was added. Images were taken and representative pictures in 10x are shown. (**B**) The contraction of this already formed tubular network was quantified 2 h (white bars) and 4 h (black bars) after FXa addition, *n* = 4, * Statistically significant difference *p* < 0.05. (**C**) HUVECs were treated with FXa, fixed and stained with Phalloidin-rhodamine to observe endothelial phenotype and stress fiber content. (**D**) Cell area was quantified using ImageJ software, *p* < 0.05. Scale bar: 10 μm.

Although we did not find evidence that FXa was delivering a survival or growth advantage to the cancer cell, we hypothesized that FXa independently from thrombin could activate endothelial cells and thus promote cancer cell metastasis. As a first approximation, changes in endothelial cell phenotype were analyzed in response to FXa. First, we used an angiogenesis assay culturing HUVECs in 3D matrigel wells; under these conditions endothelial cells form tubular structures (Figure 3A) at both 20 nM (physiological range) and 130 nM (the concentration of FXa injected into the tail vein in previous figures). Interestingly, a 2 hour-incubation with FXa causes a significant reduction in the percentage of endothelial tubular linked network at both concentrations (Figure 3B). This effect can be seen in real-time in the following video link using the endothelial cell line EA.hy926 which demonstrated the same effects as HUVEC cells (http://www.labowen.cl/cgi-bin/ Website/Pages/Internal.html.pl?PagId=51&). In this video it can be seen that individual endothelial cells elongate to form intercellular connections and the presence of FXa causes a dramatic and rapid contraction, causing the cells to return to their original form upon seeding onto Matrigel. Furthermore, these assays were performed in serum free medium and thus the observed effect was due solely to the presence of FXa, furthermore the zymogen (FX) did not show differences compared to the control (shown in Supplementary Figure S2). Exogeneous thrombin also had a similar effect. Given that FXa (and thrombin) is known to cleave and activate protease activated receptors, we included peptide agonists of Protease-activated receptor-1 and -2 (PAR-1 and PAR-2) in these experiments. As observed in Supplementary Figure S2, a PAR-1 agonist can mimic the endothelial cell contraction observed with FXa. Neither FVIIa which is known to leave PAR-2 or the PAR-2 agonist peptide showed activity in this assay. Furthermore, to demonstrate that the protease activity of FXa is required, the use of a FXa protein blocked at the active site (HCXA-EGR) showed no activity on endothelial cell contraction (Supplementary Figure S2 panels C,D) in either EA.hy926 cells or primary cultured HUVEC cells.

**Figure 4:**
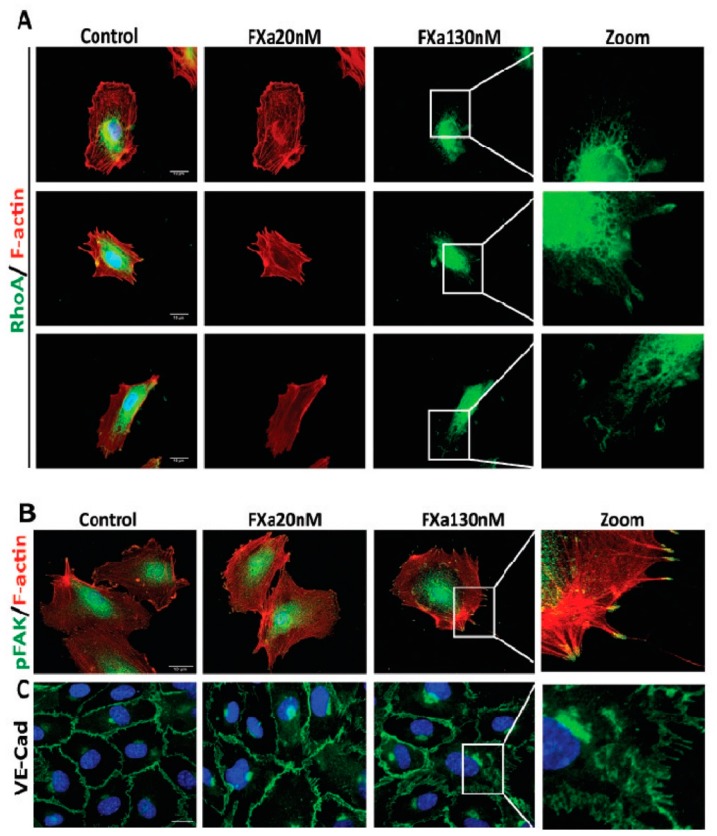
Coagulation Factor Xa promotes stress fiber formation and Vascular Endothelial-cadherin (VE-Cad) disruption. (**A**) Human Umbilical Vein Endothelial Cells (HUVECs) were treated for 5 min with FXa, fixed and prepared to the analysis of the active form of Ras homolog gene family member A (RhoA-GTP) using Immunofluorescence as described in methods. (**B**) Phosphorylated Focal Adhesion Kinase (pFAK) and VE-Cad distribution (**C**) were analyzed after 5 and 30 min of treatment respectively. Representative images from 100x and with digital zoom are shown. Scale bar: 10 μm.

Given these findings, we next assessed the response of endothelial cells at cellular level. HUVECs were seed in a high/low confluency to analyze F-actin (Figure 3C). In a high-confluency state, F-actin-rich rings are present in control conditions, however, treatment with FXa promotes the loss of F-actin-rich rings and an increase in stress fiber formation. Moreover, in a low-confluency state, there is a loss on the leading edge (lamellipodia) and an increment of F-actin enriched stress fiber formation in response to FXa. A decrease in the cell area was observed, suggesting that cell contraction was occurring (Figure 3D). The same findings were obtained in the endothelial cell line EA.hy926. Additionally, the active form of RhoA (RhoA-GTP), a small GTPase identified as a regulator of the actin cytoskeleton and implicated in endothelial hyperpermeability, was observed in highly F-actin-enriched zones surrounding the edge of cell membrane in response to FXa treatment (Figure 4A). Furthermore, phosphorylation of Focal Adhesion Kinase (pFAK, Y397) was increased in response to FXa after 5 min of treatment (Figure 4B). Consequently, FXa at both concentrations induces the disruption of VE-cadherin distribution within a confluent HUVEC monolayer (Figure 4C) and in the Human Pulmonary Microvasculature Endothelial cell line, HPMEC-1 (Figure S3), suggesting that FXa can change endothelial permeability. It is important to note that all these effects were due to FXa, as prothrombin or Thrombin were not present in this in vitro system.

### 3.3. FXa Induces Endothelial Hyperpermeability

**Figure 5:**
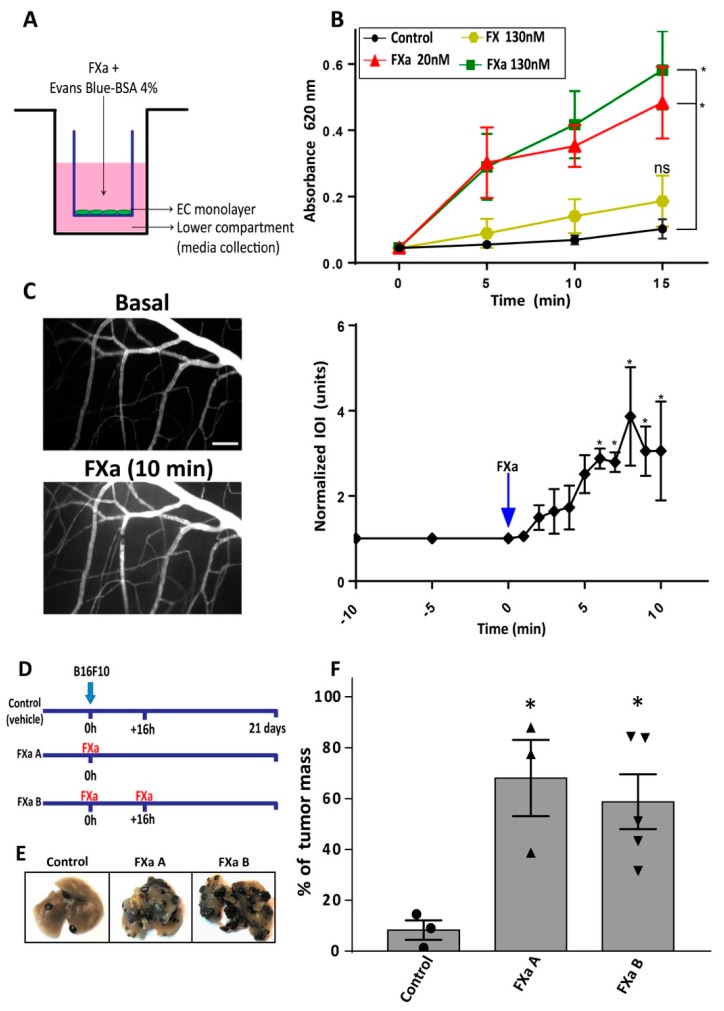
Coagulation Factor Xa induces endothelial hyperpermeability in vivo and in vitro and increases metastasis in a short-term treatment (**A**) Endothelial cells were seeded onto pre-coated Transwell (3 µm) until reach 100% confluent monolayer. Cells were treated with FXa or FX (zymogen) for 30 min in serum free media (**A**). Evans Blue-BSA was added onto the upper chamber, and 100 µL aliquots were taken from the lower chamber. (**B**) Evans Blue-BSA was measured as an indicator of hyperpermeability (Absorbance at 620 nm) in time, *n* = 5. (**C**) Cremaster muscle from mice was exposed, prepared and treated topically with FXa. Vascular hyperpermeability to Fluorescein isothiocyanate (FITC)-Dextran was evaluated using intravital microscopy and quantifying the integrated optical intensity (IOI) in the tissue, *n* = 3, *p* < 0.05. (**D**) Schematic representation of the regimens of FXa administration in mice. FXa A & B groups were treated with FXa immediately after cancer cell injection but only FXa B group received a reinforcement 16 h later. (**E**) Representative images lungs after 21 days post-injection are shown and (**F**) the quantification of tumor mass referred to total lung mass was assessed. * *p* < 0.05 was considered a statistically significant difference.

The effect of FXa treatment on an endothelial monolayer was evaluated using the Transwell system (Figure 5A). FXa, but not the zymogen FX, increases the permeability to Evans Blue-BSA (Merck, NY, USA) (Figure 4B). 

Furthermore, the effect of FXa on vascular permeability to macromolecules was analyzed in vivo using the cremaster model in C57BL/6 mice. The mouse cremasteric muscle was exposed, prepared and treated topically with FXa. Mice were perfused with Fluorescein isothiocyanate (FITC) Dextran to identify blood vessels and analyze the extravasation of this dye. After 6 min of treatment, FXa significantly increased the FITC-dextran extravasation to interstitial tissue (Figure 5C).

The aforementioned results suggest that FXa is increasing endothelial cell permeability, thus allowing cancer cells to extravasate. Therefore, we hypothesized that FXa when applied only during the first 16 h after cancer cell injection, when the cancer cells are attaching and crossing the endothelium into the pulmonary tissue, would result in increased metastasis 21 days later. Repeating the experiment detailed in Figure 2, but now applying FXa only after injection of the cancer cells and/or 16 h later (see schematic in Figure 5D), we see a clear increase in cancer metastasis at 21 days (Figure 5E,F). Our results suggest that endothelial permeability is a potential mechanism by which FXa increases metastasis in this model. 

### 3.4. FXa Changes mRNA Expression within Endothelial Cells 

**Figure 6:**
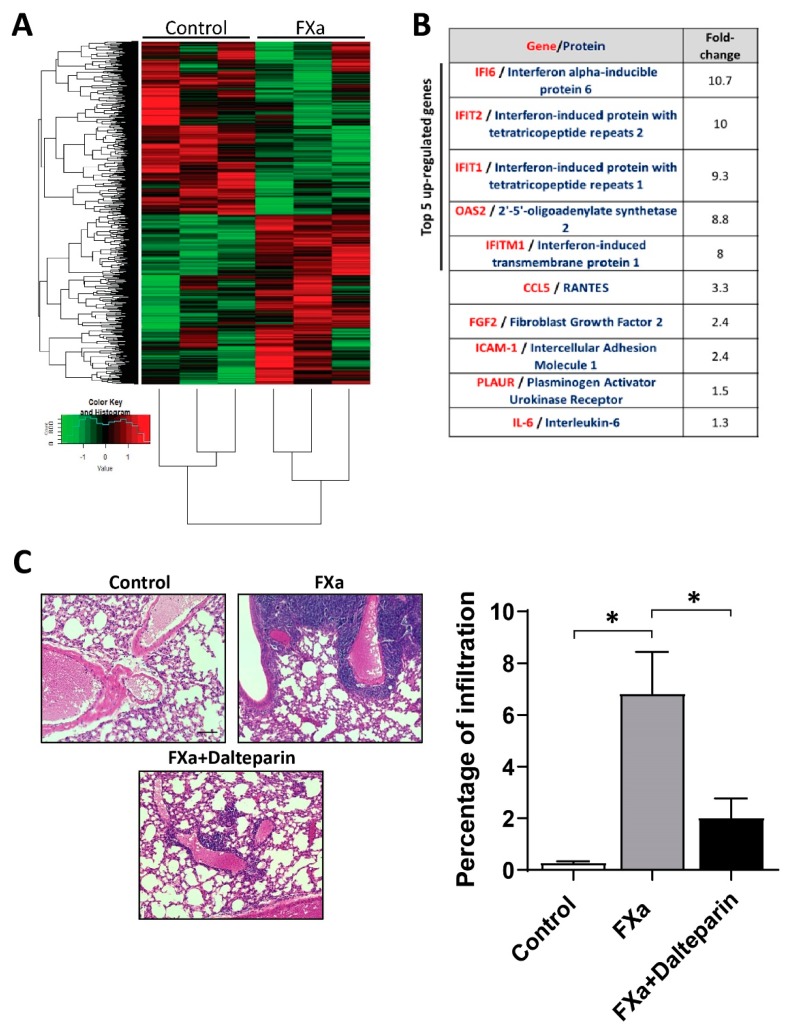
Coagulation Factor Xa treatment promotes a pro-inflammatory response. (**A**) Gene expression signature of endothelial cells treated with FXa was evaluated with the Affymetrix Human Genome 2.0 ST Array. Heatmap shows the 6 analyzed samples (control vs treatment with FXa130nM for 4 h) clustered by condition at the left side of the image. Red and green colors represent up and downregulated genes respectively, and a short list of the up-regulated genes is shown in panel (B). (**C**) Lungs from mice treated with Vehicle, FXa and FXa+Dalteparin were stained with H&E to analyze immune cell infiltration. Scale bar 100 μm. (**D**) Immune infiltration was quantified and expressed as the percentage of total area per lung, * *p* < 0.05.

Given the change in endothelial phenotype promoted by FXa, we assessed changes at the transcriptional level using a GeneChip Human Gene 2.0 ST Array (Thermofisher, Carlsband, CA, USA). After 4 h of treatment (EA.hy926 endothelial cells), 127 genes were significantly up-regulated and 36 were down-regulated in response to FXa. These differences between treatments are represented in a heatmap (Figure 6A). A protein interaction network was generated based on the data from up-regulated genes and using the Gene Ontology Consortium database, showing a main cluster of proteins associated with the Interferon (IFN) response pathway (Supplementary Figure S4). Transcripts of proteins associated with angiogenesis (Fibroblast Growth Factor-2, FGF-2), inflammation (Interleukin 6, (IL-6) and ICAM-1), extracellular matrix degradation (Plasminogen Activator Urokinase Receptor, PLAUR) and immune chemokines (CCL5) were also up-regulated in response to FXa (Figure 6B). In accordance with the microarray result, analysis of the non-cancer containing sections of the mice lungs (from experimental procedures used in Figure 2) demonstrated that FXa treatment invoked an inflammatory response (Figure 6C). As detected by the H&E stain (Abcam, San Francisco, CA, USA), an immune infiltration after FXa treatment was observed principally close to large blood vessels and was reversed or prevented by the presence of the anticoagulant dalteparin.

### 3.5. FXa Increases Cancer Cell Adhesion to the Endothelium

**Figure 7:**
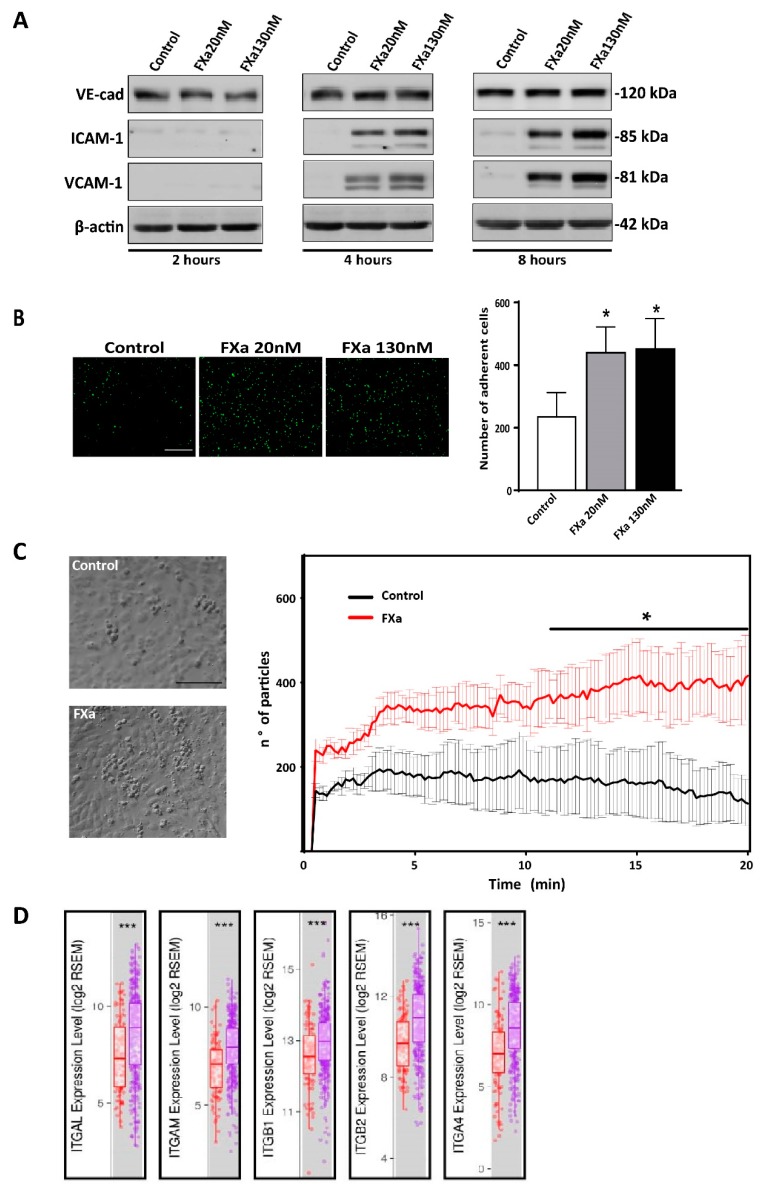
Coagulation Factor Xa promotes adhesion molecules expression in Human Umbilical Vein Endothelial Cells (HUVECs) and increases the adhesion of B16F10 under static/flow conditions. (**A**) HUVECs were treated with FXa and total protein levels were analyzed. Representative images of 4 independent HUVEC primary cultures are shown. (**B**) B16F10-eGFP cells were seeded for 30 min over a confluent monolayer of HUVEC cells pretreated with either vehicle or FXa for 4 h. Subsequently, cells were washed and fixed and the number of B16F10-eGFP cells that remained attached were determined by fluorescence microscopy, *n* = 4 *p* < 0.05. * was considered a statistically significant difference. Scale bar 100 μm. (**C**) A microfluidic adhesion assay, where HUVEC cells were treated for 6 h with either control of FXa (130 nM) prior to underflow conditions (flow 50 µL/min) delivering 175,000 B16F10 cells per minute. Resulting adherent cells were evaluated with live imaging for 20 min and then quantified using ImageJ software *n* = 3 *p* < 0.05 * was considered a statistically significant difference. Scale bar 50 μm. (**D**) The Cancer Genome Atlas-Tumor IMmune Estimation Resource (TCGA-TIMER) database was used to address the relative RNA expression of endothelial adhesion molecules ligands (red boxplot represents primary tumors from melanoma patients and purple boxplots represent metastasis samples). Data was extracted from 333 primaries and 331 metastatic melanomas patients (https://cistrome.shinyapps.io/timer/). *** represents a statistically significant difference with *p* < 0.01.

Prior to cancer cell extravasation during metastasis, cancer cells must be attached firmly to the endothelium which allows them to withstand the pressure delivered by blood flow. As adhesion molecules allow cancer cell capture at the endothelial wall prior to extravasation, we assessed whether HUVEC increased adhesion molecules when treated with FXa. Figure 7A shows that FXa increased the protein levels of ICAM-1 and VCAM-1 after 4 and 8 h of treatment. Changes in VE-Cadherin levels were not detected. Pre-treatment of a confluent monolayer of HUVEC with FXa for 4 h significantly increased the number of attached B16F10-eGFP cancer cells under static conditions (Figure 7B). Finally, to assess an increase of adhesion under physiological conditions, we used a microfluidic microchannel where B16F10 cells were pumped over a FXa pre-treated HUVEC monolayer. In line with our previous experiment, we observed that under flow conditions FXa increased adhesion of B16F10 to endothelial cells (Figure 7C). Consistent with an important role of cell adhesion molecules ICAM-1 and VCAM-1 in melanoma metastasis, using the Tumor IMmune Estimation Resource (TIMER) using the The Cancer Genome Atlas (TCGA) database, we observed there is a significant increase of the adhesion molecules ligands: integrin alpha L chain (ITGAL), Integrin subunit alpha M (ITGAM), Integrin subunit beta 1 (ITGB1), Integrin subunit beta 2 (ITGB2) and integrin alpha subunit (ITGA4) RNA levels in primary tumor vs metastasis (Figure 7D). 

## 4. Discussion

A recognized relationship between the coagulation system and cancer progression is now in its second century of investigation, yet the exact participation of the coagulation factors upon the metastatic process is still poorly understood. The publications generated in hematology suggest that the coagulation factors possess yet-to-defined functions upon the intact endothelium and in tumor microenvironment. Herein, we present evidence that activated coagulation factor X can promote solid tumor volume and experimental metastasis. In this paper, we concentrated on the mechanism of action solely in the B16F10 metastasis model. We demonstrated decreased melanoma metastasis in the presence of the FXa inhibitor dalteparin and thus open the door to the evaluation of direct FXa inhibitors in the exclusive setting of cancer metastasis. Dalteparin is a low molecular weight heparin which in complex with antithrombin inhibits FXa in an allosteric manner, by binding to an exosite [41]. It will be interesting to examine, using chloromethylketone derivatives, whether this allosteric blockage of FXa is responsible for the reduced in vivo pro-metastatic effects of FXa. Our observations in vitro that activation of PAR-1 also mimics FXa-mediated endothelial contraction, and furthermore that the failure of active-site-blocked FXa to cause contraction, suggests that the protease activity of FXa is involved in this process. The mechanism by which elevated FXa causes increased solid tumor volume also requires further investigation. In relation to this question, we did note in our experiments that (as with the B16F10 cells) FXa did not have alter cell cycle or migration of the human cancer cell lines. Herein, we present evidence that the action of FXa is not upon the cancer cell, but in fact upon the vascular endothelium, resulting in adhesion molecules expression and hyperpermeability, thus potentially promoting the process of extravasation (metastasis). Interestingly, the use of anticoagulants has long been associated with improved cancer patient outcome, potentially through the decrease of thrombotic events however the positive non-hemostatic effect of an anticoagulant regime has not yet been addressed [26]. Despite the FRAGMATIC trial using the preferential FXa inhibitor dalteparin in cancer patients being established to evaluate this, no beneficial action upon cancer patient survival was observed [27]. However, we hypothesize that in this trial, the lack of a beneficial effect occurred because most of enrolled patients that had already presented metastasis, thus, a possibly beneficial effect of the anticoagulant over cancer metastasis and progression may have been lost or diluted out from the study. After half a century of cancer investigation it is now clear that every cancer stage is unique, and the same compound may promote or inhibit cancer progression depending on the cancer stage. Three oral FXa inhibitors: apixaban, rivaroxaban and edoxaban, have passed clinical trials and are US Food and Drug Administration approved for the treatment of Venous Thromboembolism (VTE) in cancer patients. A potential anti-cancer effect of these second-generation anticoagulants is under evaluation. However, as we hypothesize that the coagulation cascade leading to a blood clot is independent to the action of coagulation factors upon the endothelium, it is a possibility that anticoagulants such as apixaban, rivaroxaban or dabigatran (thrombin inhibitor) may have differing effects upon cancer metastasis than the results we observed with Dalteparin. A recent publication has reported that neither rivaroxaban or dabigatran reduce metastasis in vivo mouse models [42], while rivaroxaban has been shown to increase neovascularization [43]. The non-hemostatic effect of coagulation proteases (such as: FVIIa, FXa, thrombin and Activated Protein C) are intimately linked to PAR-1 and PAR-2 signaling. We show that endothelial cell contraction can be mediated by a PAR-1, but not PAR-2, agonist peptide. These receptors are widely expressed among several cell types and triggering different responses. Platelet activation (through PAR-1 cleavage) allows the attachment to the endothelium via von Willebrand Factor-Glycoprotein Ib interaction, causing the release of several molecules that regulate wound healing and inflammation [44]. The binding of coagulation proteases to the PARs receptors is regulated by conserved regions like the Gla domain and Epidermal growth factor-like domains. Interestingly, Feistritzer and collaborators proposed that FXa had a barrier-protective role into endothelial monolayers through PAR-1 and -2 activation [45], a process dependent on the binding to the endothelial protein C receptor [46]. PAR1 signaling on endothelial cells promotes G Protein coupling leading to Protein Kinase C (PKC), Ca^2+^, Nuclear Factor κappa β (NFκβ) levels and RhoA activation and subsequently endothelial barrier disruption [47]. Our immunofluorescent results suggest that the RhoA pathway is activated in response to FXa, and thus we speculate that the endothelial cell contraction could be triggered by myosin light chain phosphorylation leading to our observed stress fiber formation. Moreover, morphological and functional observations in vivo and in vitro suggest that FXa, independently of Thrombin, have anti-angiogenic properties over vein-derived (HUVEC) endothelial cells and this is mediated by PAR-1 but not PAR-2 [48]. Recently, it has been proposed that alterations in the permeability of the endothelium occur in response to the activation of PAR-2 by FXa in arterial and microvascular cells in vitro and this is independent of the TF/FVIIa complex [49]. While we clearly demonstrate specific actions of FXa in in vitro/in vivo models and we show an effect of the predominantly FXa inhibitor dalteparin over metastasis, our study has caveats. Although our in vitro FXa experiments were performed in the absence of prothrombin and thrombin, certain cancer promoting effects of FXa in vivo may contain a contribution from thrombin. This possibility is hard to control. The zymogen FX (endotoxin free) that was isolated under the same methodology and applied in an identical manner did not demonstrate activity in our experiments. Given the dual and potentially independent action of coagulation factors upon endothelial permeability and coagulation, the use of exclusive thrombin-inhibitors or other anticoagulants may independently affect cancer metastasis. Experiments are currently ongoing to address this issue and determine the role of the new generation of anticoagulants in this melanoma metastasis model. Despite the uncertainty that the increase in cancer metastasis may be aided in part by thrombin generation, our work clearly shows that activation of the coagulation system promotes metastasis, and this can be reduced by a first-line treatment anticoagulant such as the predominantly FXa-inhibitor dalteparin. 

Chronic inflammation is a known contributor to metastasis and inflammatory cells have been shown to contribute to the pre-metastatic niche [50]. Moreover, molecules such as Histamine, Tumor Necrosis Factor alpha (TNF-α), Lipopolysaccharide (LPS), are known to promote endothelial hyperpermeability and adhesion molecule exposure, suggesting a potent role over cancer metastasis [51]. As an example, LPS exposure, and consequential inflammatory response, results in increased B16F10 lung metastasis [52]. Herein, we demonstrate by microarray analysis that pro-inflammatory signals, principally the interferon response pathway, are enhanced within the endothelium upon FXa exposure and this was confirmed by increased immune cell infiltration in FXa-treated mouse lungs. In accordance with our results, other authors have reported elevated IL-6 and IL-8 release from HUVEC in response to FXa [15]. FXa has also been reported to increase monocyte attachment to the endothelium and our microarray also demonstrated an increase in ICAM-1. Promisingly, dalteparin reduced immune cell infiltration into the lung, offering another potentially beneficial clinical action of this drug [53]. Using a similar model, reduced leukocyte infiltration into mouse lung was observed by other authors upon the intravenous introduction of the coagulation factor VIIa after prior exposure to LPS [31]. We also observed in our microarray that PLAUR, which is associated with extracellular matrix remodeling and the metastatic process, was upregulated. Furthermore, FXa has been reported to stimulate metalloproteases from vascular smooth muscle cells and thus may be helping metastasis process in vivo [54]. Whether the increase in lung metastasis observed by FXa in our model is an effect of an inflammatory response or occurs as a consequence of tumor cell invasion will be part of future experiments and investigation. However, this result clearly demonstrates the association between the coagulation cascade activation, endothelial activation, inflammation and metastasis. 

Interestingly, the cancer promoting action of this coagulation factor was not restricted to metastasis. We demonstrate that the presence of FXa increased the growth of solid tumors in two distinct immunocompromised mice models injected with human cell lines (Figure 1). While in this paper we concentrated on the role of FXa upon the endothelial activation, and the potential increase in metastasis due to enhanced extravasation, it did not escape our intention that the presence of FXa on a continual basis caused a greater increase in lung tumor mass in the B16F10 model than that brought about by initial dosage. This may suggest, and hopefully future experiments will demonstrate, that FXa also provides a growth advantage to already established tumor masses of B16F10 cells, whether it be in the mouse lung or flank. As we observed no effect of FXa upon biological actions (cell cycle, migration etc.) of cancer any of the three cancer cell lines used (Supplementary Figure S1), we can speculate that the inflammatory environment enhanced by FXa is promoting tumor growth and/or there is a promotion of vascular growth to irrigate the tumor mass. 

## 5. Conclusions

In our mouse models, increased circulating FXa promotes tumor burden and melanoma metastasis. Our data suggest that the mechanism of action of FXa involves a direct effect upon endothelial cells. Given the observed inflammatory response profile observed in our microarray, the NFκβ pathway is likely to be involved in the overexpression of the adhesion molecules shown in our figures. FXa increases ICAM and VCAM on the endothelial cells, this in turn leads to enhanced adhesion of cancer cells and potentially increased extravasation occurs due to an increase in cytoskeletal rearrangement and endothelial permeability. Our data, together with limited information from clinical trials, suggest beneficial effects of anticoagulant treatment, especially dalteparin and low-molecular-weight heparins, in the setting of metastatic melanoma disease. We propose that future trials should have the premise that not all anticoagulants will have the same outcomes, nor will all stages of the malignant process show a beneficial response to anticoagulant treatment. This and other approaches may finally deliver a beneficial clinical application to Armand Trousseau’s 1865 observation that cancer and hypercoagulability are connected. 

## Supplementary Materials

The following are available online at https://www.mdpi.com/2072-6694/11/8/1103/s1. Figure S1: Coagulation Factor Xa does not alter cancer cell biology in vitro, Figure S2: Coagulation Factor Xa and Protease activated Protein-1 (PAR-1) peptide agonist cause contraction of endothelial cells forming. tubular structures, Figure S3: Coagulation Factor Xa promotes Vascular Endothelial-Cadherin disruption in the Human Pulmonary Microvasculature Endothelial Cells, HPMEC-1, Figure S4: A protein interaction network generated based on the data from up-regulated genes and using the Gene Ontology Consortium database.

## References

[1] Noble J., Pasi J. (2010). Epidemiology and pathophysiology of cancer-associated thrombosis. Br. J. Cancer.

[2] Elyamany G., Alzahrani A.M., Bukhary E. (2014). Cancer-associated thrombosis: An overview. Clin. Med. Insights Oncol..

[3] Falanga A., Marchetti M., Vignoli A. (2013). Coagulation and cancer: Biological and clinical aspects. J. Thromb. Haemost..

[4] Desch A., Gebhardt C., Utikal J., Schneider S.W. (2017). D-dimers in malignant melanoma: Association with prognosis and dynamic variation in disease progress. Int. J. Cancer..

[5] Sparsa A., Durox H., Doffoel-Hantz V., Munyangango E.M., Bédane C., Cendras J., Gantois C., Boulinguez S., Bonnetblanc J.M. (2011). High prevalence and risk factors of thromboembolism in stage IV melanoma. J. Eur. Acad. Dermatol. Venereol..

[6] Kasthuri R.S., Taubman M.B., Mackman N. (2009). Role of tissue factor in cancer. J. Clin. Oncol..

[7] Henriquez S., Calderon C., Quezada M., Oliva B., Bravo M.L., Aranda E., Kato S., Cuello M.A., Gutierrez J., Quest A.F. (2011). Progesterone utilizes distinct membrane pools of tissue factor to increase coagulation and invasion and these effects are inhibited by TFPI. J. Cell Physiol..

[8] Krupiczojc M.A., Scotton C.J., Chambers R.C. (2008). Coagulation signalling following tissue injury: Focus on the role of factor Xa. Int. J. Biochem. Cell Biol..

[9] Haddad T.C., Greeno E.W. (2006). Chemotherapy-induced thrombosis. Thromb. Res..

[10] Kirwan C.C., Mccollum C.N., Mcdowell G., Byrne G.J. (2015). Investigation of Proposed Mechanisms of Chemotherapy-Induced Venous Thromboembolism: Endothelial Cell Activation and Procoagulant Release Due to Apoptosis. Clin. Appl. Thromb..

[11] Laresche C., Pelletier F., Garnache-Ottou F., Lihoreau T., Biichlé S., Mourey G., Saas P., Humbert P., Seilles E., Aubin F. (2014). Increased levels of circulating microparticles are associated with increased procoagulant activity in patients with cutaneous malignant melanoma. J. Investig. Dermatol..

[12] Zigler M., Kamiya T., Brantley E.C., Villares G.J., Bar-Eli M. (2011). PAR-1 and thrombin: The ties that bind the microenvironment to melanoma metastasis. Cancer Res..

[13] Han N., Jin K., He K., Cao J., Teng L. (2011). Protease-activated receptors in cancer: A systematic review. Oncol. Lett..

[14] Borensztajn K., Peppelenbosch M.P., Spek C.A. (2010). Coagulation Factor Xa inhibits cancer cell migration via LIMK1-mediated cofilin inactivation. Thromb. Res..

[15] Senden N.H., Jeunhomme T.M., Heemskerk J.W., Wagenvoord R., van’t Veer C., Hemker H.C., Buurman W.A. (1998). Factor Xa Induces Cytokine Production and Expression of Adhesion Molecules by Human Umbilical Vein Endothelial Cells. J. Immunol..

[16] Ebrahimi S., Rahmani F., Behnam-Rassouli R., Hoseinkhani F., Parizadeh M.R., Keramati M.R., Khazaie M., Avan A., Hassanian S.M. (2017). Proinflammatory signaling functions of thrombin in cancer. J. Cell. Physiol..

[17] Nguyen D.X., Bos P.D., Massagué J. (2009). Metastasis: from dissemination to organ-specific colonization. Nat. Rev. Cancer.

[18] Läubli H., Borsig L. (2010). Selectins as Mediators of Lung Metastasis. Cancer Microenviron..

[19] Bendas G., Borsig L. (2012). Cancer cell adhesion and metastasis: Selectins, integrins, and the inhibitory potential of heparins. Int. J. Cell Biol..

[20] Ferjancic Š., Gil-Bernabé A.M., Hill S.A., Allen P.D., Richardson P., Sparey T., Savory E., McGuffog J., Muschel R.J. (2013). VCAM-1 and VAP-1 recruit myeloid cells that promote pulmonary metastasis in mice. Blood.

[21] Kolaczkowska E., Kubes P. (2013). Neutrophil recruitment and function in health and inflammation. Nat. Rev. Immunol..

[22] Vestweber D. (2008). VE-cadherin: The major endothelial adhesion molecule controlling cellular junctions and blood vessel formation. Arterioscler. Thromb. Vasc. Biol..

[23] Lampugnani M.G., Dejana E. (2007). Adherens junctions in endothelial cells regulate vessel maintenance and angiogenesis. Thromb. Res..

[24] Reymond N., D’Água B.B., Ridley A.J. (2013). Crossing the endothelial barrier during metastasis. Nat. Rev. Cancer.

[25] van Nieuw Amerongen G.P., van Delft S., Vermeer M.A., Collard J.G., van Hinsbergh V.W. (2012). Activation of RhoA by Thrombin in Endothelial Hyperpermeability. Circ. Res..

[26] Gerotziafas G.T., Papageorgiou C., Hatmi M., Samama M.M., Elalamy I. (2008). Clinical studies with anticoagulants to improve survival in cancer patients. Pathophysiol. Haemost. Thromb..

[27] Griffiths G.O., Burns S., Noble S.I., Macbeth F.R., Cohen D., Maughan T.S. (2009). FRAGMATIC: A randomised phase III clinical trial investigating the effect of fragmin^®^ added to standard therapy in patients with lung cancer. BMC Cancer.

[28] Altinbas M., Coskun H.S., Er O., Ozkan M., Eser B., Unal A., Cetin M., Soyuer S. (2004). A randomized clinical trial of combination chemotherapy with and without low-molecular-weight heparin in small cell lung cancer. J. Thromb. Haemost..

[29] Kakkar A.K., Levine M.N., Kadziola Z., Lemoine N.R., Low V., Patel H.K., Rustin G., Thomas M., Quigley M., Williamson R.C. (2004). Low molecular weight heparin, therapy with dalteparin, and survival in advanced cancer: The fragmin advanced malignancy outcome study (FAMOUS). J. Clin. Oncol..

[30] Albadawi H., Witting A.A., Pershad Y., Wallace A., Fleck A.R., Hoang P., Khademhosseini A., Oklu R. (2017). Animal models of venous thrombosis. Cardiovasc. Diagn. Ther..

[31] Kondreddy V., Wang J., Keshava S., Esmon C.T., Rao L.V.M., Pendurthi U.R. (2018). Factor VIIa induces anti-inflammatory signaling via EPCR and PAR1. Blood.

[32] Torres-Estay V., Carreño D.V., Fuenzalida P., Watts A., San Francisco I.F., Montecinos V.P., Sotomayor P.C., Ebos J., Smith G.J., Godoy A.S. (2016). Androgens modulate male-derived endothelial cell homeostasis using androgen receptor-dependent and receptor-independent mechanisms. Angiogenesis.

[33] Oyarce C., Cruz-Gomez S., Galvez-Cancino F., Vargas P., Moreau H.D., Diaz-Valdivia N., Diaz J., Salazar-Onfray F.A., Pacheco R., Lennon-Dumenil A.M. (2017). Caveolin-1 expression increases upon maturation in dendritic cells and promotes their migration to lymph nodes thereby favoring the induction of CD8+ T cell responses. Front. Immunol..

[34] Patterson C.E., Rhoades R.A., Garcia J.G. (1992). Evans blue dye as a marker of albumin clearance in cultured endothelial monolayer and isolated lung. J. Appl. Physiol..

[35] Marín N., Zamorano P., Carrasco R., Mujica P., González F.G., Quezada C., Meininger C.J., Boric M.P., Durán W.N., Sánchez F.A. (2012). S-Nitrosation of β-catenin and p120 catenin: A novel regulatory mechanism in endothelial hyperpermeability. Circ. Res..

[36] Durán W.N., Seyama A., Yoshimura K., González D.R., Jara P.I., Figueroa X.F., Borić M.P. (2000). Stimulation of NO production and of eNOS phosphorylation in the microcirculation in vivo. Microvasc. Res..

[37] Bustos M.A., Lucchesi O., Ruete M.C., Mayorga L.S., Tomes C.N. (2012). Rab27 and Rab3 sequentially regulate human sperm dense-core granule exocytosis. Proc. Natl. Acad. Sci. USA.

[38] Erices R., Cubillos S., Aravena R., Santoro F., Marquez M., Orellana R., Ramírez C., González P., Fuenzalida P., Bravo M.L. (2017). Diabetic concentrations of metformin inhibit platelet-mediated ovarian cancer cell progression. Oncotarget.

[39] Palta S., Saroa R., Palta A. (2014). Overview of the coagulation system. Indian J. Anaesth..

[40] Bunce M.W., Toso R., Camire R.M. (2011). Zymogen-like factor Xa variants restore thrombin generation and effectively bypass the intrinsic pathway in vitro. Blood.

[41] Rezaie A.R. (2000). Heparin-binding exosite of factor Xa. Trends Cardiovasc. Med..

[42] Buijs J.T., Laghmani E.H., van den Akker R.F.P., Tieken C., Vletter E.M., van der Molen K.M., Crooijmans J.J., Kroone C., Le Dévédec S.E., van der Pluijm G. (2019). The Direct Oral Anticoagulants Rivaroxaban and Dabigatran do not Inhibit Orthotopic Growth and Metastasis of Human Breast Cancer in Mice. J. Thromb. Haemost..

[43] Wu T.C., Chan J.S., Lee C.Y., Leu H.B., Huang P.H., Chen J.S., Lin S.J., Chen J.W. (2015). Rivaroxaban, a factor Xa inhibitor, improves neovascularization in the ischemic hindlimb of streptozotocin-induced diabetic mice. Cardiovasc. Diabetol..

[44] Golebiewska E.M., Poole A.W. (2015). Platelet secretion: From haemostasis to wound healing and beyond. Blood Rev..

[45] Feistritzer C., Lenta R., Riewald M. (2005). Protease-activated receptors-1 and -2 can mediate endothelial barrier protection: Role in factor Xa signaling. J. Thromb. Haemost..

[46] Schuepbach R.A., Riewald M. (2010). Coagulation factor Xa cleaves protease-activated receptor-1 and mediates signaling dependent on binding to the endothelial protein C. receptor. J. Thromb. Haemost..

[47] Artim-Esen B., Smoktunowicz N., McDonnell T., Ripoll V.M., Pericleous C., MacKie I., Robinson E., Isenberg D., Rahman A., Ioannou Y. (2017). Factor Xa Mediates Calcium Flux in Endothelial Cells and is Potentiated by Igg from Patients with Lupus and/or Antiphospholipid Syndrome. Sci. Rep..

[48] Lange S., Gonzalez I., Pinto M.P., Arce M., Valenzuela R., Aranda E., Elliot M., Alvarez M., Henriquez S., Velasquez E.V. (2014). Independent Anti-Angiogenic Capacities of Coagulation Factors, X. and Xa. J. Cell Physiol..

[49] Benelhaj N.E., Maraveyas A., Featherby S., Collier M.E.W., Johnson M.J., Ettelaie C. (2019). Alteration in endothelial permeability occurs in response to the activation of PAR2 by factor Xa but not directly by the TF-factor VIIa complex. Thromb. Res..

[50] Liu Y., Cao X. (2016). Characteristics and Significance of the Pre-metastatic Niche. Cancer Cell.

[51] Pober J.S., Sessa W.C. (2007). Evolving functions of endothelial cells in inflammation. Nat. Rev. Immunol..

[52] Tang Y.M., Cao Q.Y., Guo X.Y., Dong S.H., Duan J.A., Wu Q.N., Liang Q.L. (2018). Inhibition of p38 and ERK1/2 pathways by Sparstolonin B suppresses inflammation-induced melanoma metastasis. Biomed. Pharmacother..

[53] Natoni A., Macauley M.S., O’Dwyer M.E. (2016). Targeting Selectins and Their Ligands in Cancer. Front. Oncol..

[54] Dass K., Ahmad A., Azmi A.S., Sarkar S.H., Sarkar F.H. (2008). Evolving role of uPA/uPAR system in human cancers. Cancer Treat. Rev..

