# Supplementary material for: Coagulation Factor Xa Promotes Solid Tumor Growth, Experimental Metastasis and Endothelial Cell Activation
Source: Cancers (Basel). 2019 Aug 2;11(8):1103. doi: 10.3390/cancers11081103 (PMC6721564; doi:10.3390/cancers11081103)
Supplement: Supplementary file 1 [file cancers-11-01103-s001.pdf]

Supplemetray Materials

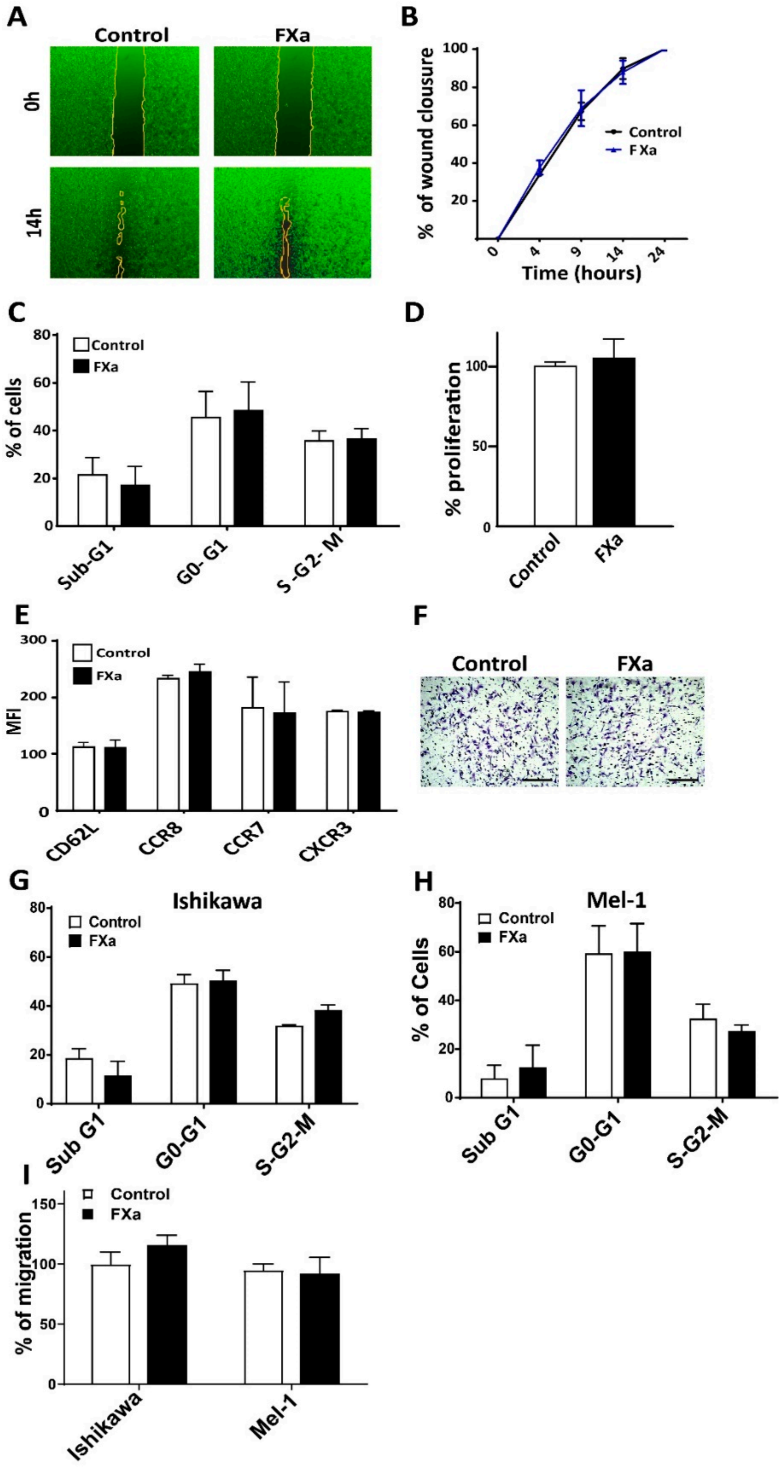

Figure S1. Coagulation Factor Xa does not alter cancer cell biology in vitro. (**A**) A scratch was generated upon a 100% confluent B16F10 cell monolayer. FXa or vehicle addition was added to the medium and wound closure was evaluated at varying time points up to 24 hours. Representative images at time zero and 14

hours are shown in (**A**) and the rate of closure overtime in (**B**) *n* = 3, $p > 0.05$. (**C**) Cells were treated with FXa for 24 hours, then fixed and stained with propidium iodide. Cell cycle was analyzed using flow cytometry, *n* = 3, $p > 0.05$. (**D**) Semi-confluent B16F10 were treated with FXa for 24h hours and proliferation evaluated using the colorimetric reagent WST-1, *n* = 3, $p > 0.05$. (**E**) The adhesion molecule CD62L and the chemokines receptors CCR8, CCR7 and CXCR3 levels in cancer cells were analyzed by flow cytometry in response to FXa treatment, *n* = 3, $p > 0.05$. (**F**) B16F10 were treated with vehicle or FXa for 24 hours prior to seeding in Matrigel containing Boyden chambers and invasion assessed after 8 hours. Cells were fixed and stained with crystal violet, scale bar 100 µm. Using the same protocol as panel C, Ishikawa (**H**) and Mel-1 (**G**) cancer cells were treated with vehicle or FXa and cell cycle was analyzed by flow cytometry at 24 hours, *n* = 3, $p > 0.05$. (I) Cell migration was evaluated in Ishikawa and Mel-1 cancer cells using Boyden chambers with 10% Fetal Bovine Serum as a chemoattractant, *n* = 2. Cell were treated with vehicle or FXa and allowed to migrate for 18 hours. Migrating cells were counted and expressed graphically as a percentage of vehicle-treated cells (set at 100%).

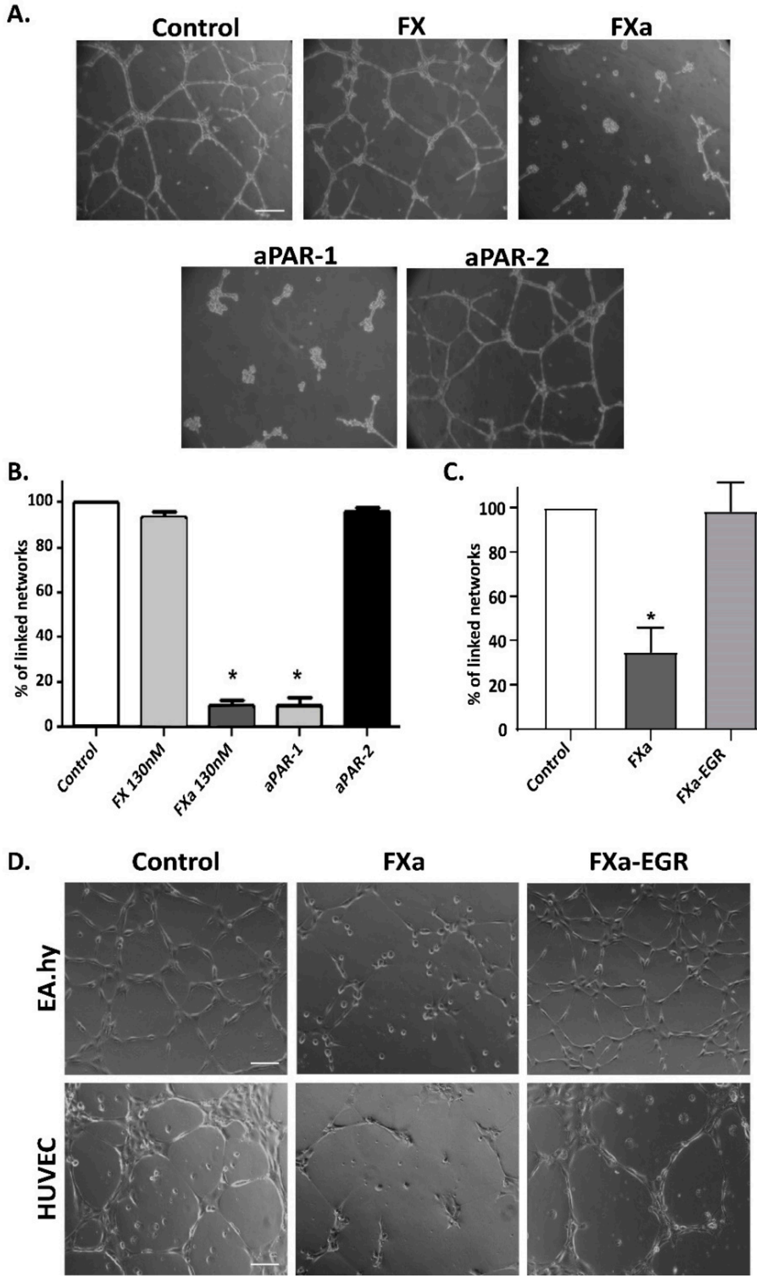

Figure S2. Coagulation Factor Xa and Protease activated Protein-1 (PAR-1) peptide agonist cause contraction of endothelial cells forming. tubular structures. EA.hy296 endothelial cells were seeded on Matrigel in the presence of Vascular Endothelial Growth Factor (10 ng/mL). Once tubular structures had formed, FX (zymogen), FXa, PAR-1 or PAR-2 peptide agonists were added. Panel (**A**) shows representative images, which are shown graphically with statistics in panel (**B**) ($n = 3$). Lower panels demonstrate that a FXa active site mutant fails to cause endothelial cell contraction (EAhy926 and HUVEC, panel, * $p < 0.05$.(**D**). Panel (**C**) represents EAhy926 tubular network quantification, $n = 3$, * $p < 0.05$. Scale bar 50 µm.

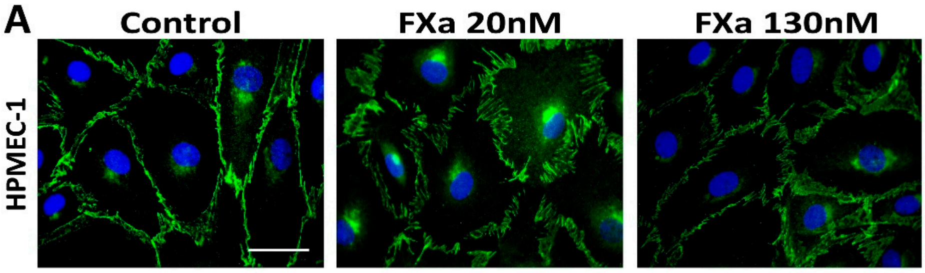

Figure S3. Coagulation Factor Xa promotes Vascular Endothelial-Cadherin disruption in the Human Pulmonary Microvasculature Endothelial Cells, HPMEC-1. Distribution of VE-cadherin pattern was analyzed after 30 minutes of treatment with either vehicle or FXa. Representative images from 100x amplification are shown. Scale bar 10 µm.

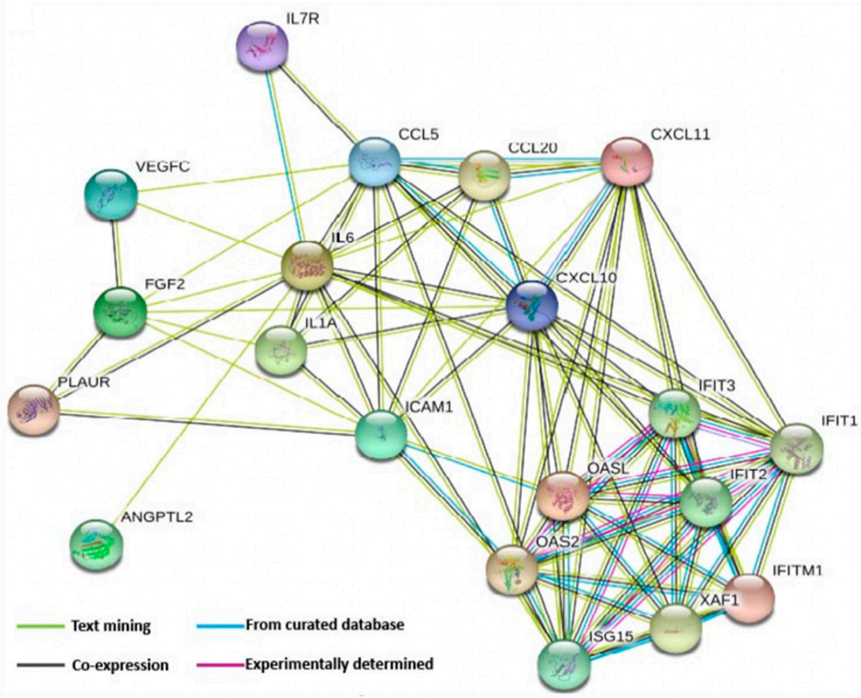

Figure S4. A protein interaction network generated based on the data from up-regulated genes and using the Gene Ontology Consortium database. The main cluster of proteins associates with the Interferon (IFN) response pathway.
